# Supplementary material for: Capturing critical gem-diol intermediates and hydride transfer for anodic hydrogen production from 5-hydroxymethylfurfural
Source: Nat Commun. 2023 Dec 18;14:8395. doi: 10.1038/s41467-023-43704-2 (PMC10728175; doi:10.1038/s41467-023-43704-2)
Supplement: Supplementary file 1 — supplementary Information [file 41467_2023_43704_MOESM1_ESM.docx]

**Supplementary Information**

Capturing Critical *gem*-Diol Intermediates and Hydride Transfer for Anodic Hydrogen Production from 5-Hydroxymethylfurfural

Guodong Fu ^1^, Xiaomin Kang ^2^, Yan Zhang ^3^, Ying Guo ^1^, Zhiwei Li ^4^, Jianwen Liu ^1*^, Lei Wang ^1^, Jiujun Zhang ^5,6^, Xian-Zhu Fu ^1*^ and Jing-Li Luo ^1*^

*^1^* Shenzhen Key Laboratory of Energy Electrocatalytic Materials, Guangdong Research Center for Interfacial Engineering of Functional Materials, College of Materials Science and Engineering, Shenzhen University, Shenzhen, 518060, China

*^2^* School of Mechanical Engineering, University of South China, Hengyang, 421001, Hunan Province, China.

*^3^* Pingshan Translational Medicine Center, Shenzhen Bay Laboratory, Shenzhen 518055, Guangdong Province, China

*^4^* National Supercomputing Center in Shenzhen, Shenzhen, 518055, China

*^5^* College of Materials Science and Engineering, Fuzhou University, Fuzhou, 350108, China

*^6^* Institute for Sustainable Energy, College of Science, Shanghai University, Shanghai, 200444, China

E-mail: Jianwen Liu: jwliu@szu.edu.cn; Xian-Zhu Fu: xz.fu@szu.edu.cn; Jing-Li Luo: jll@szu.edu.cn

**Keywords:** Anodic hydrogen production, 5-Hydroxymethylfurfural, *gem*-Diol intermediates, *In situ* Raman

**Supplementary Table 1** Vibrational peak assignment of the Raman spectra at 0.13-0.43 V.

| Vibrational peak assignment | Calculated Raman shift (cm^−1^) | | | | | | Experiment Raman shift(cm^-1^) |
| --- | --- | --- | --- | --- | --- | --- | --- |
|  | HMFMD^2−^-Au_13_ | HMFMD^−^-Au_13_ | HMFCA-Au_13_ | | H-Au_13_ | |  |
| ν(Au-O) | 428 |  | |  | |  | 412 |
| ν(Au-O) |  | 466 | |  | |  | 449 |
| ν(Au-O) |  |  | | 475 | |  | 472 |
| ν(C-O) |  | 936 | |  | |  | 945 |
| ν(C-O) | 1027 |  | | 1024 | |  | 1024 |
| ν(C=C-C=C) |  | 1537 | | 1512 | |  | 1514 |
| ν_as_(C=C-C=C) |  |  | | 1544 | |  | 1557 |
| ν(Au-H) |  |  | |  | | 1869 | 1830-1866 |

Au-O stretching peaks are significantly enhanced in the Raman spectra.

**Supplementary Table 2** Vibrational peak assignment of the Raman spectra at 0.53-0.93 V.

| Vibrational peak assignment | Calculated wavenumber (cm^−1^) | | Experiment wavenumber (cm^−1^) |
| --- | --- | --- | --- |
|  | HMFMD^−^-Au_13_ | HMFCA-Au_13_ |  |
| v(Au-O) | 466 |  | 449 |
| ν(Au-O) |  | 475 | 472 |
| ν(C-O) | 936 |  | 947 |
| ν(C-O) | 973 |  | 969 |
| ν(C-O) |  | 1024 | 1023 |
| ν(C-O) | 1066 |  | 1060 |
| ν(C=C-C=C) | 1512 | 1538 | 1512 |
| ν(C=C-C=C) |  | 1543 | 1566 |

Au-O stretching peaks are significantly enhanced in the Raman spectra.


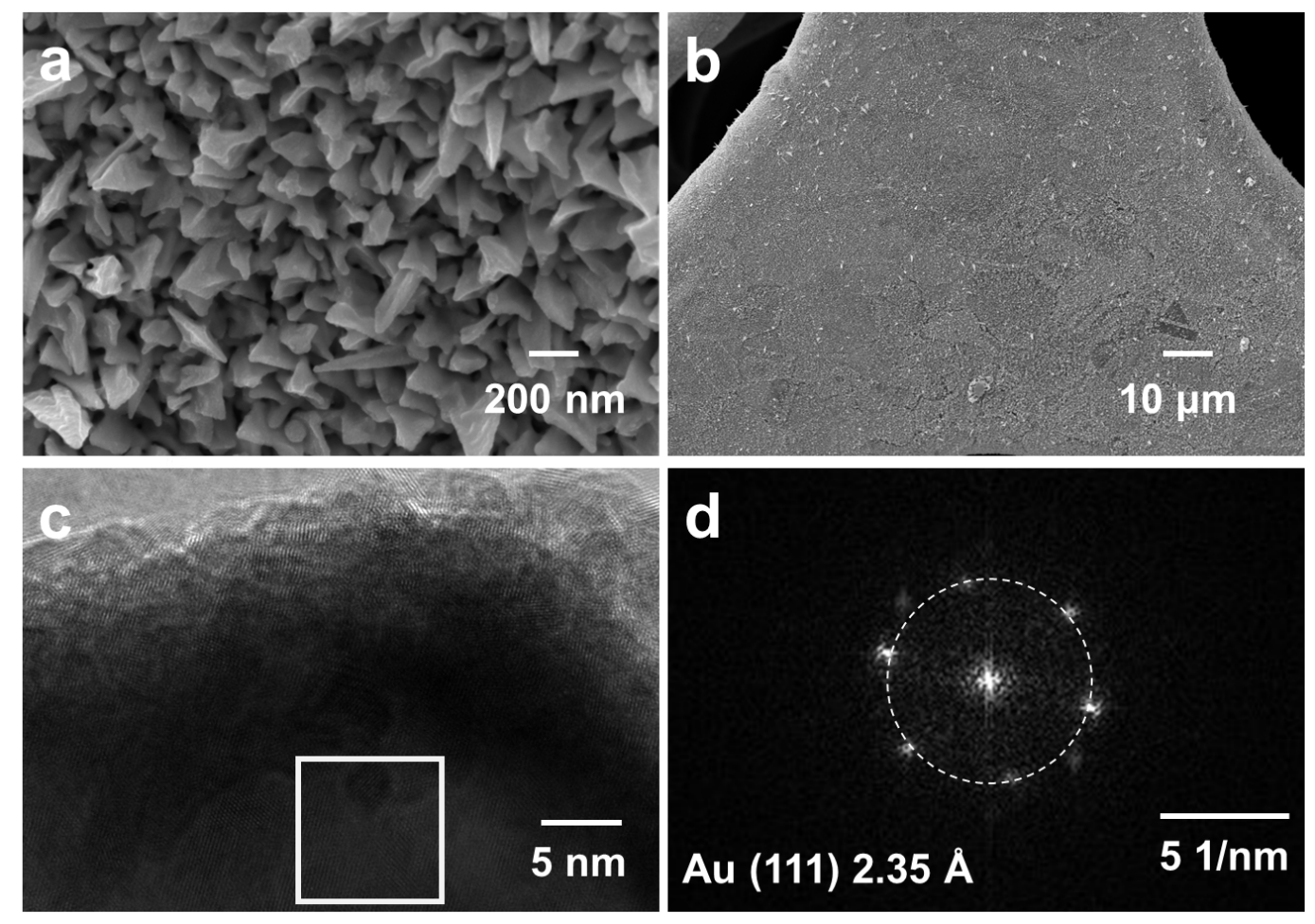


**Supplementary Figure 1** (a-b) SEM images of an Au-Ni electrode (c) TEM image of the Au-Ni electrode surface, and (d) Fourier transform image of the selected area (white square area) in the TEM image.


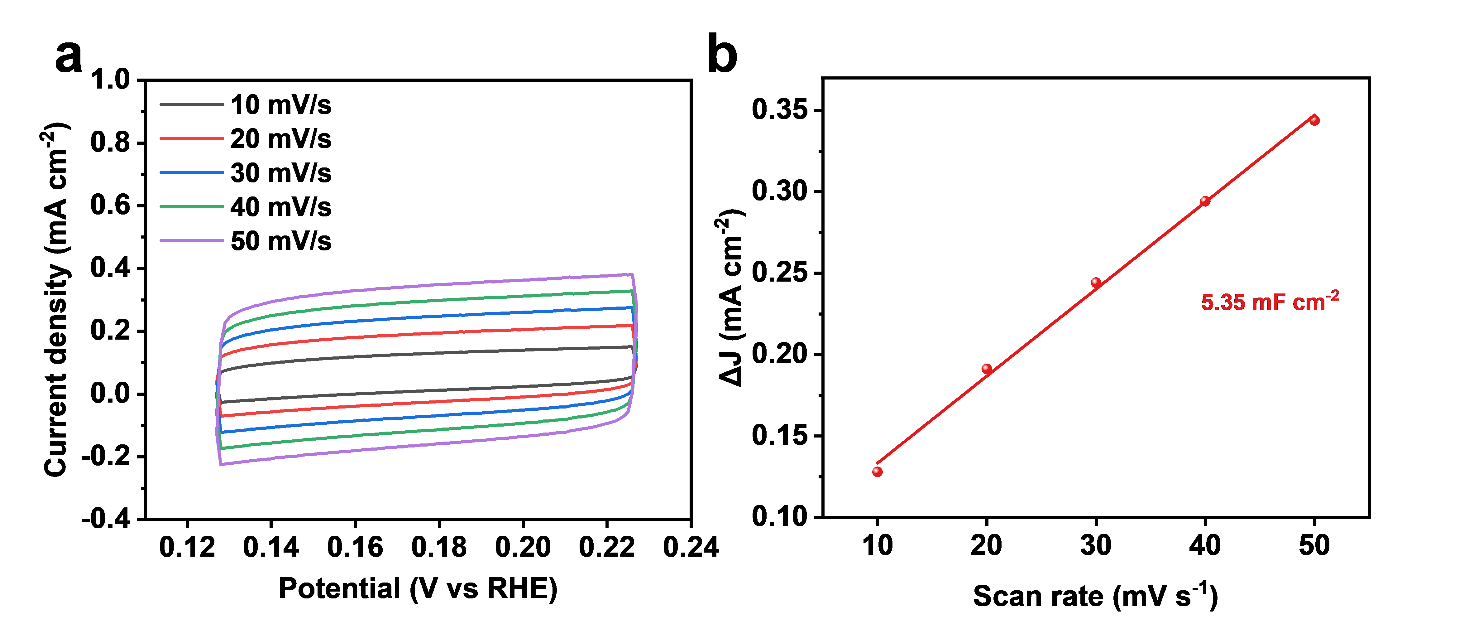


**Supplementary Figure 2** (a) CV curve of Ni-Au electrode in 1 M KOH. (b) Linear fitting of the C_dl_ for Au electrode.


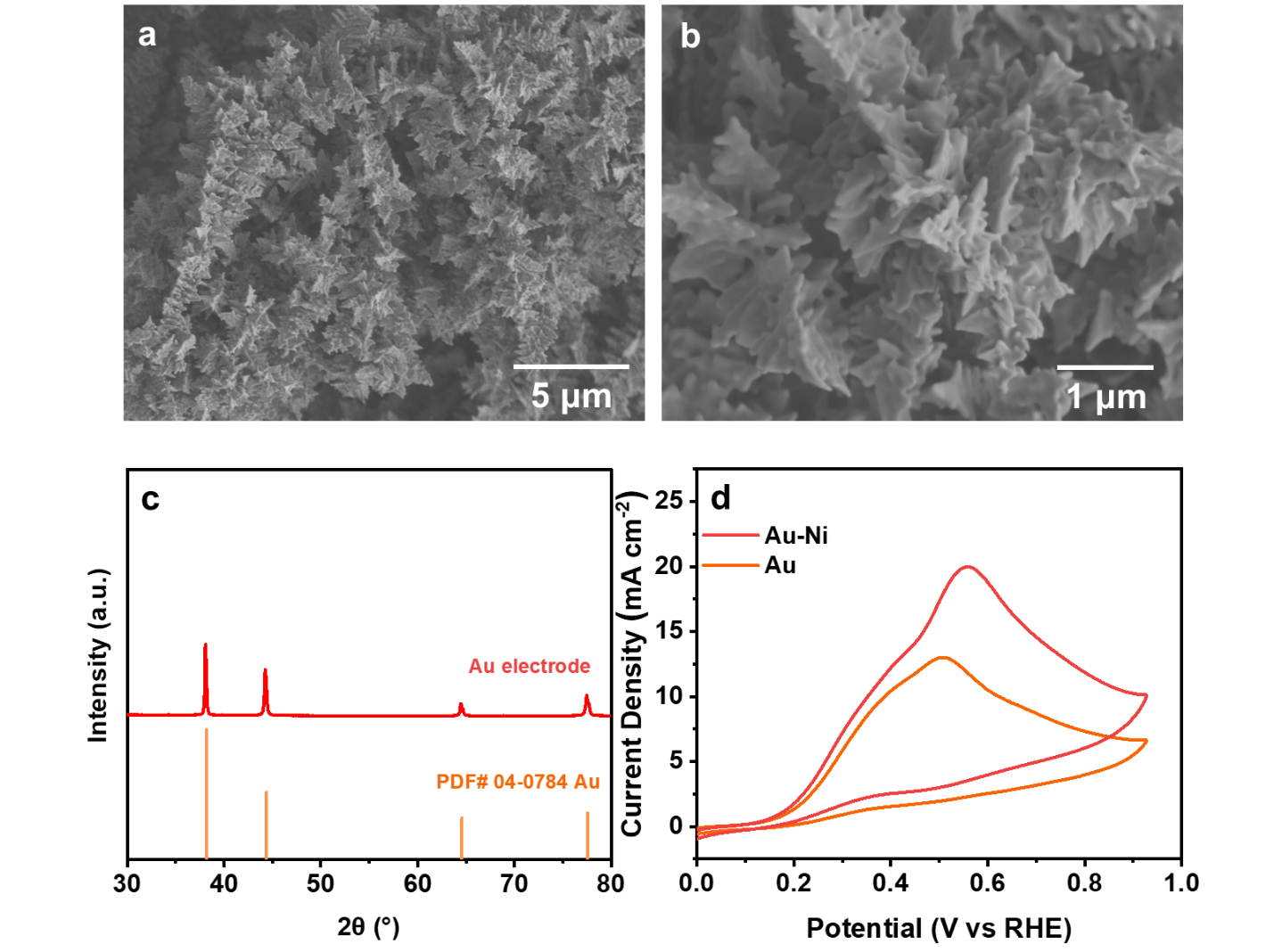


**Supplementary Figure 3** SEM images of as synthesis Au nanostructure electrode in low (a) and high (b) magnification. (c) The XRD pattern of Au nanostructure electrode. (d) The LSV curve of Au nanostructure electrode in 1 M KOH and 0.05 M HMF.


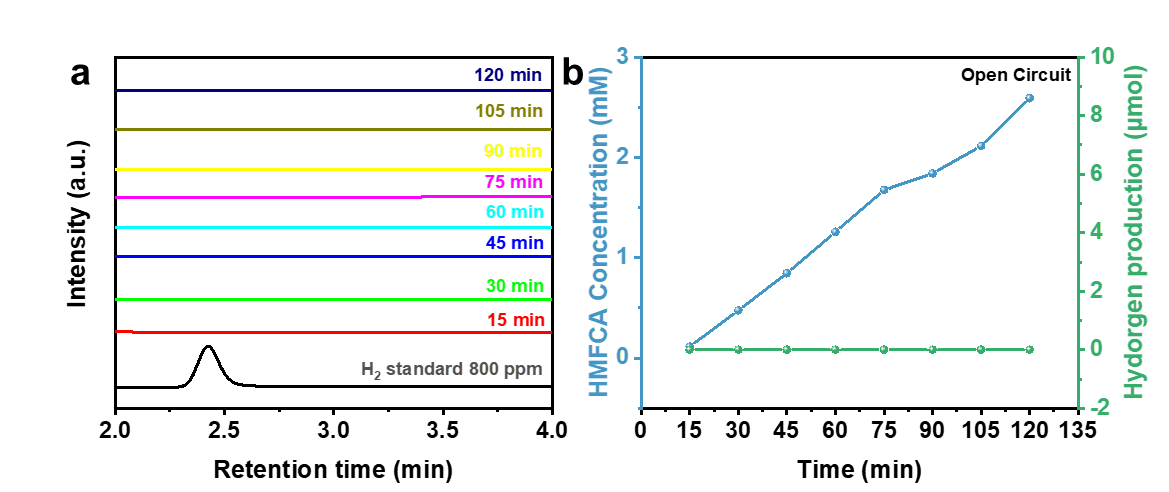


**Supplementary Figure 4** (a) GC curve from 1 M KOH and 0.05 M HMF without applied potential. (b) Yield of HMFCA and H_2_ in 1 M KOH and 0.05 M HMF without applied potential.


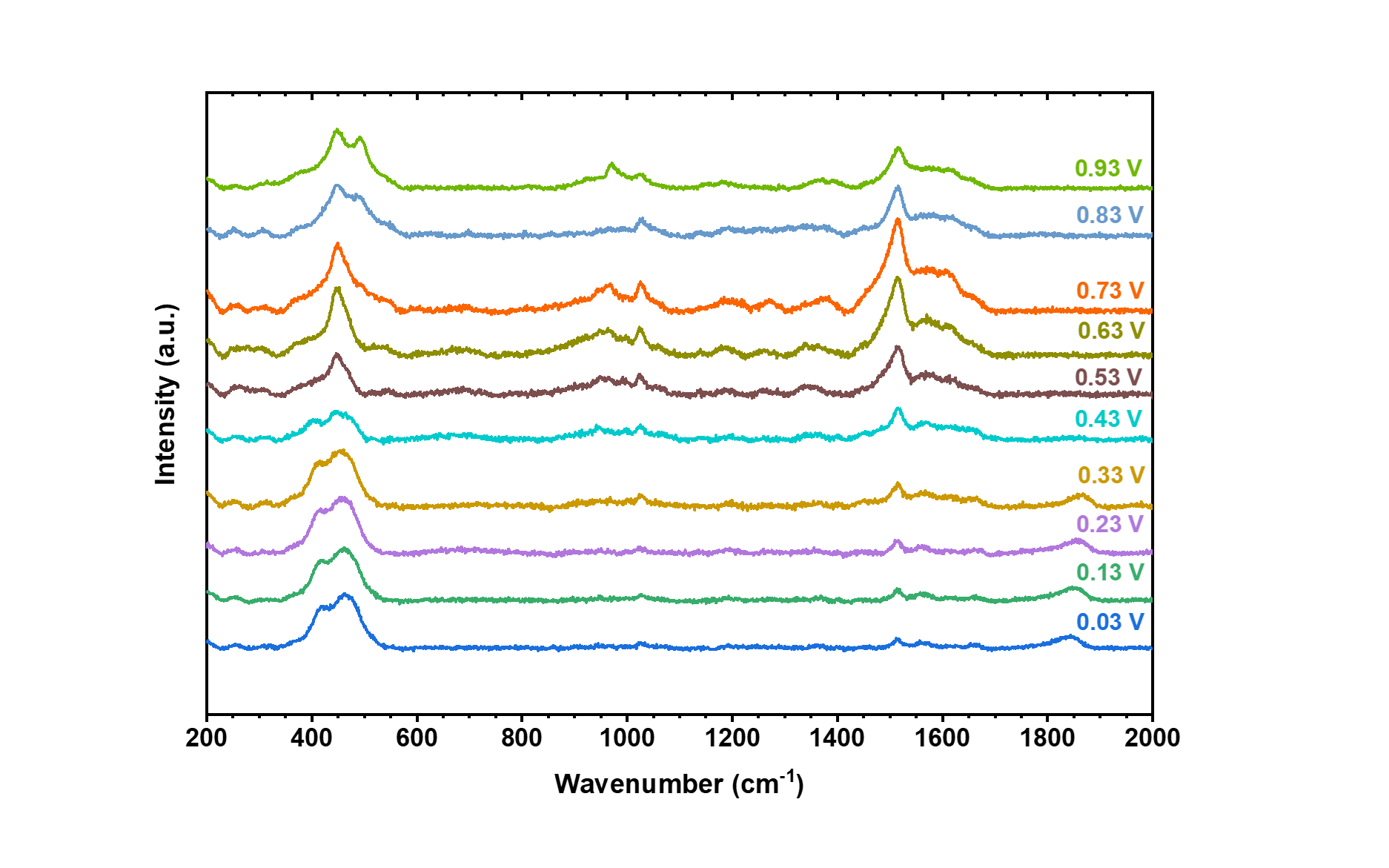


**Supplementary Figure 5** *In situ* Raman spectra of HMF oxidation reaction on gold at 1M KOH 0.05 M HMF.

**
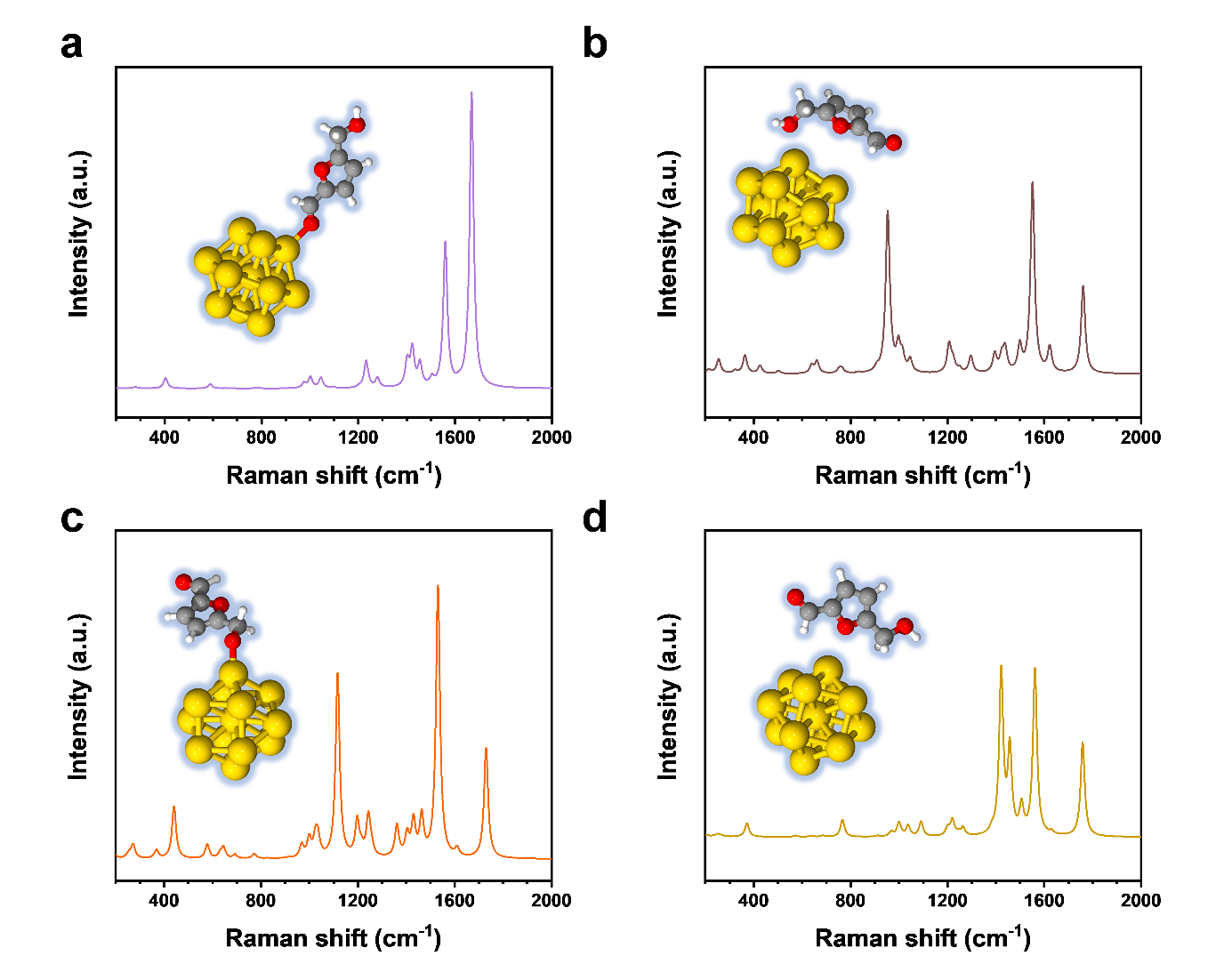
**

**Supplementary Figure 6** Calculated Raman spectra of HMF adsorption on Au_13_ with different absorption group: a) aldehyde absorption, b) hydroxyl absorption, c) alkoxyl absorption and d) furan-O absorption.

**
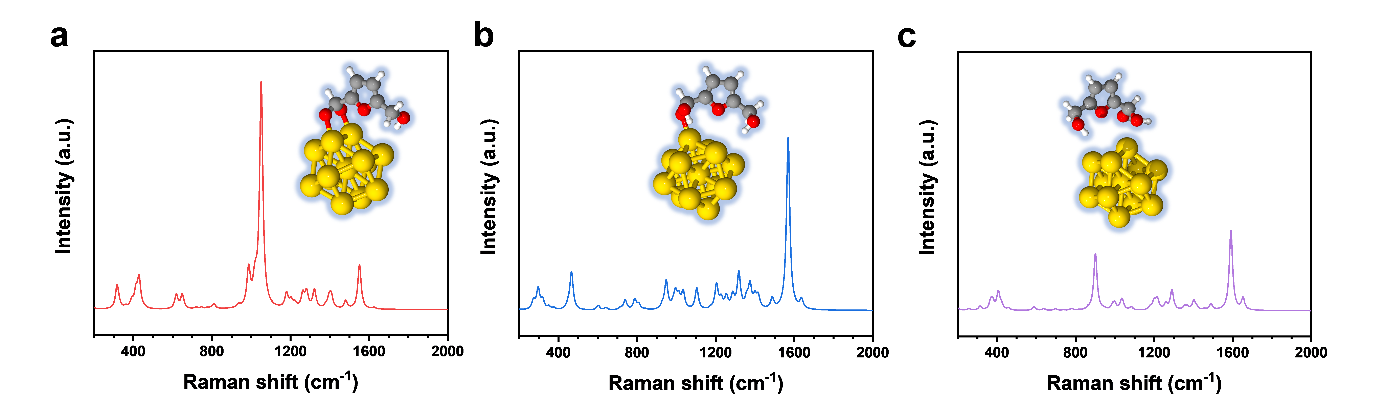
**

**Supplementary Figure 7** Calculated Raman spectra of (a) HMFMD^2−^-Au_13_, (b) HMFMD^−^-Au_13_ and (c) HMFMD-Au_13_

**
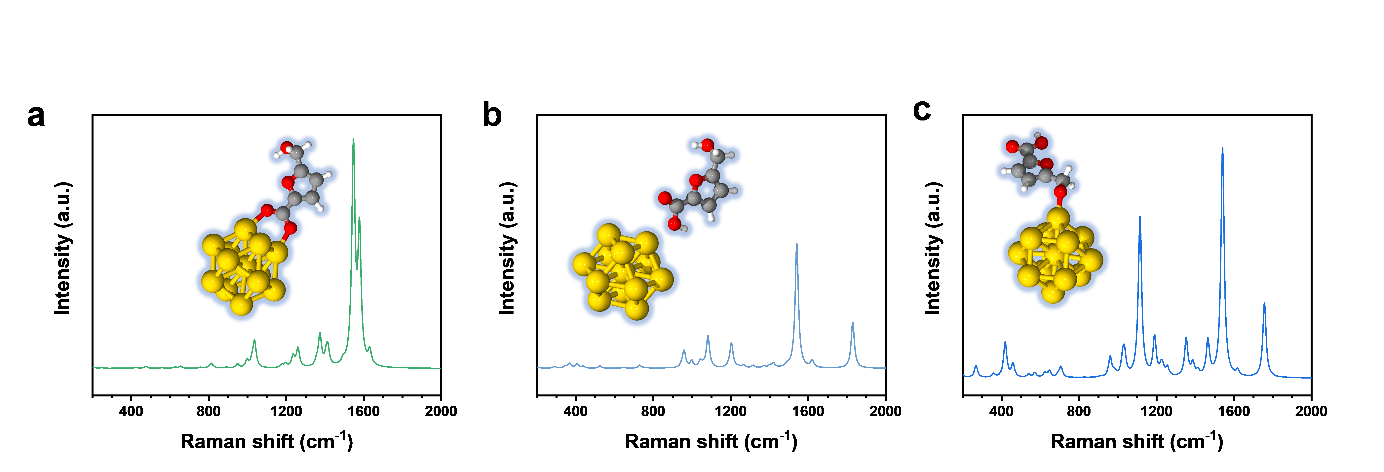
**

**Supplementary Figure 8** Calculated Raman spectra of HMFCA adsorption on Au_13_ with different absorption group: (a) carboxyl anion absorption, (b) carboxyl absorption and (c) alkoxyl absorption.


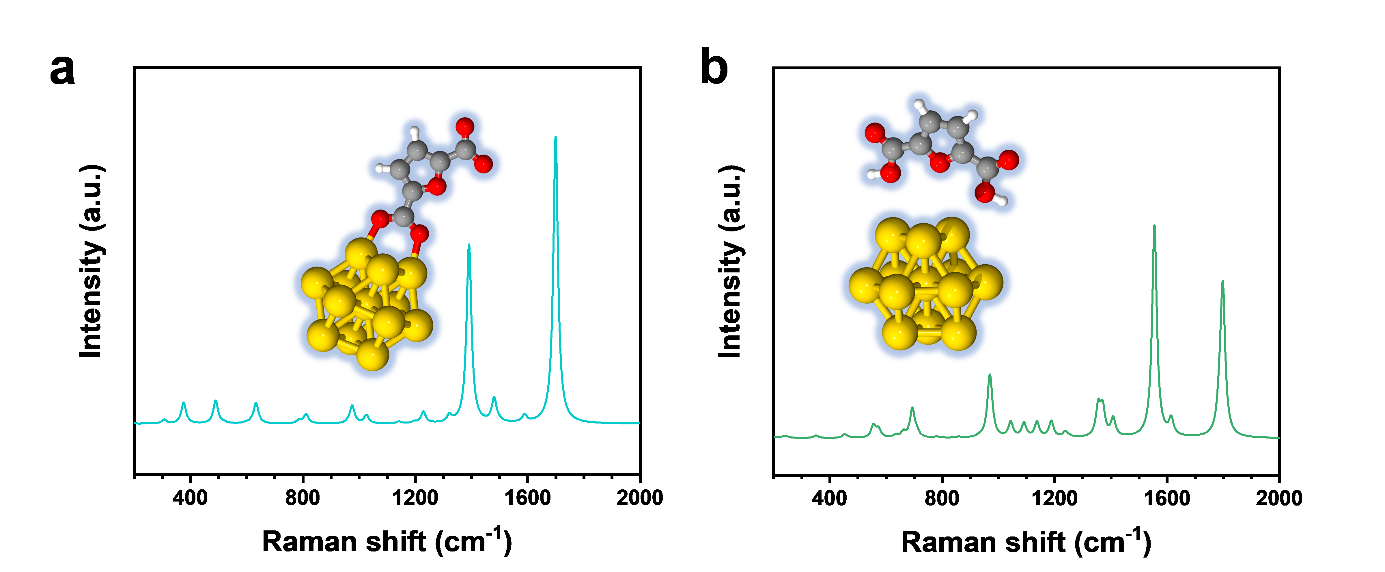


**Supplementary Figure 9** Calculated Raman spectra of FDCA adsorption on Au_13_ with different absorption group: (a) carboxyl anion absorption, (b) carboxyl absorption.


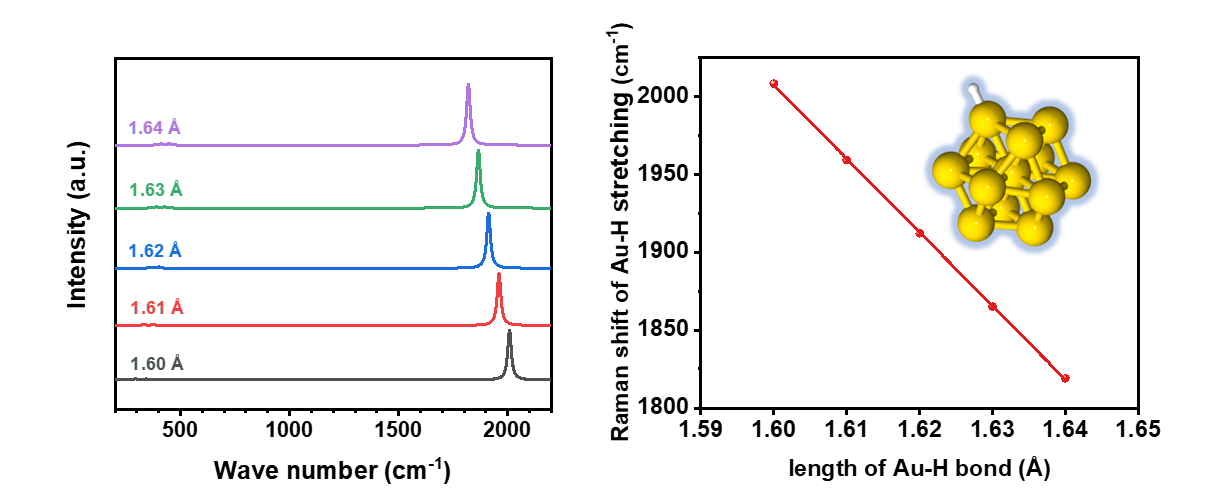


**Supplementary Figure 10** (a) Calculated Raman spectra of H-Au_13_ with different length of Au-H bond. (b) Linear fitting for the calculated Raman shift and length of Au-H bond.


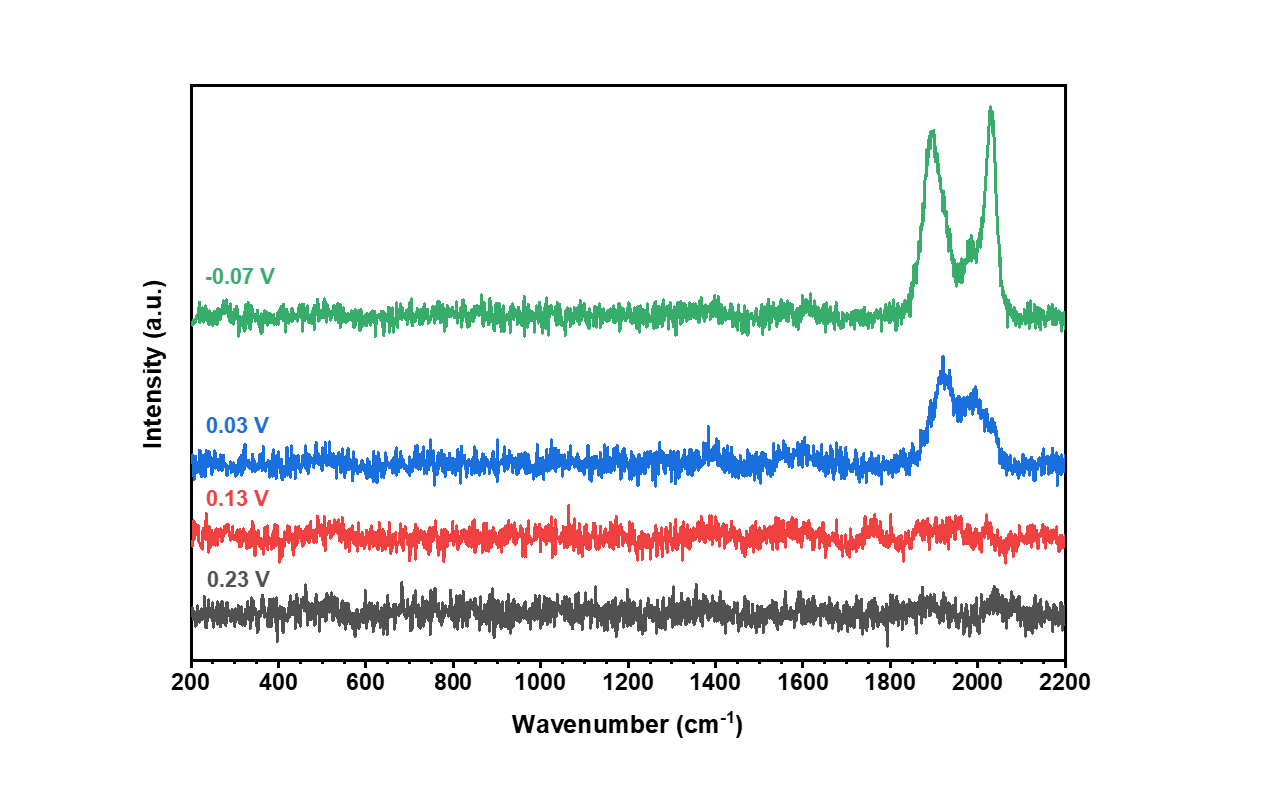


**Supplementary Figure 11** *In situ* Raman spectra of hydrogen evolution reaction on gold at 1M KOH.


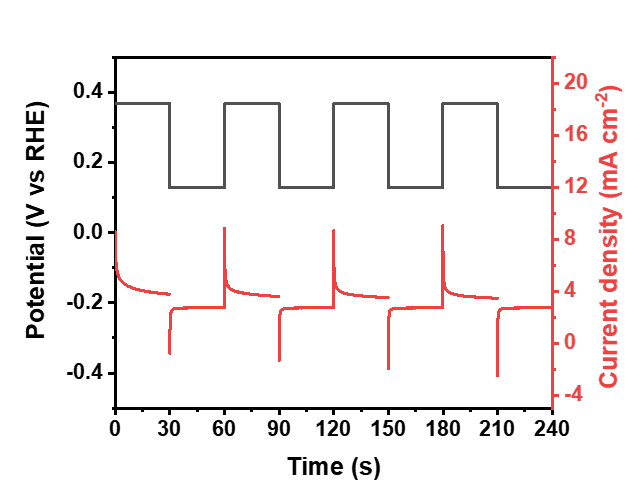


**Supplementary Figure 12** Potential and current signal of HMF oxidation reaction on Au electrode in 1 M KOD and 0.5 M HMF using D2O as the solvent at 0.33V.


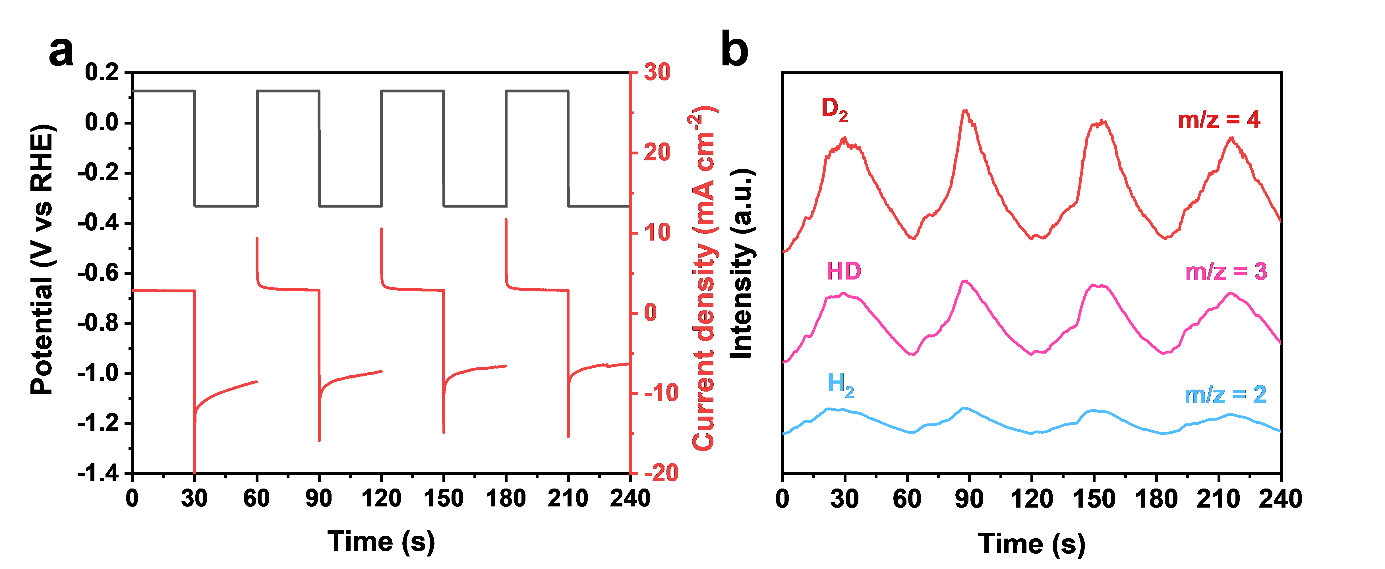


**Supplementary Figure 13 (**a) Potential and current signal and (b) Differential Electrochemical Mass Spectrometry (DEMS) signal of hydrogen evolution reaction on Au electrode in 1 M KOD and 0.5 M HMF using D_2_O as the solvent at −0.37V.

**
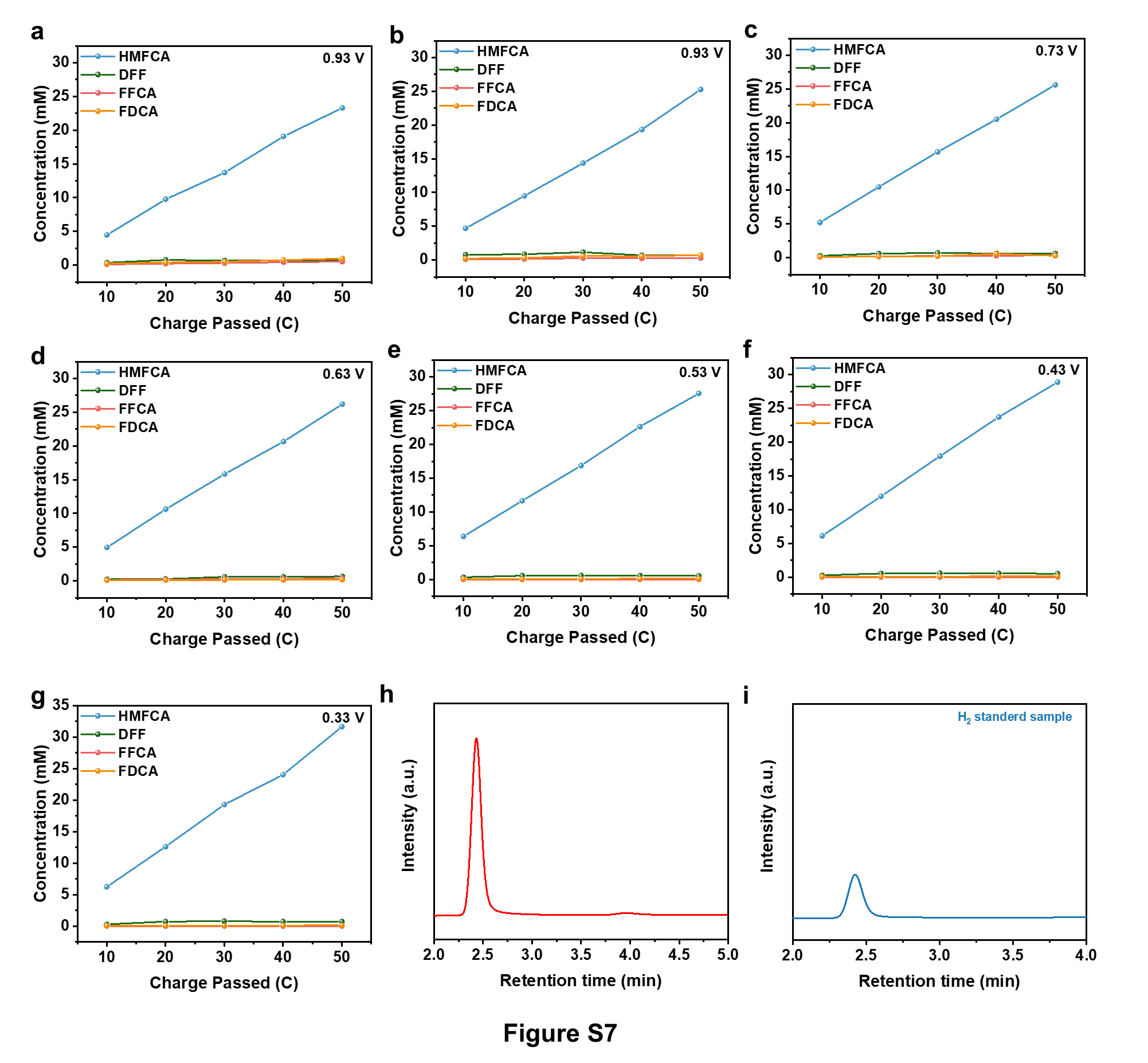
**

**Supplementary Figure 14** Concentration of detected products during HMF oxidation reaction at (a) 0.93 V, (b) 0.83 V, (c) 0.73 V, (d) 0.63 V, (e) 0.53V, (f) 0.43 V and **(**g) 0.33 V. Gases chromatogram traces of (h) the gases from HMF oxidation reaction and (i) H_2_ standered gas.

**
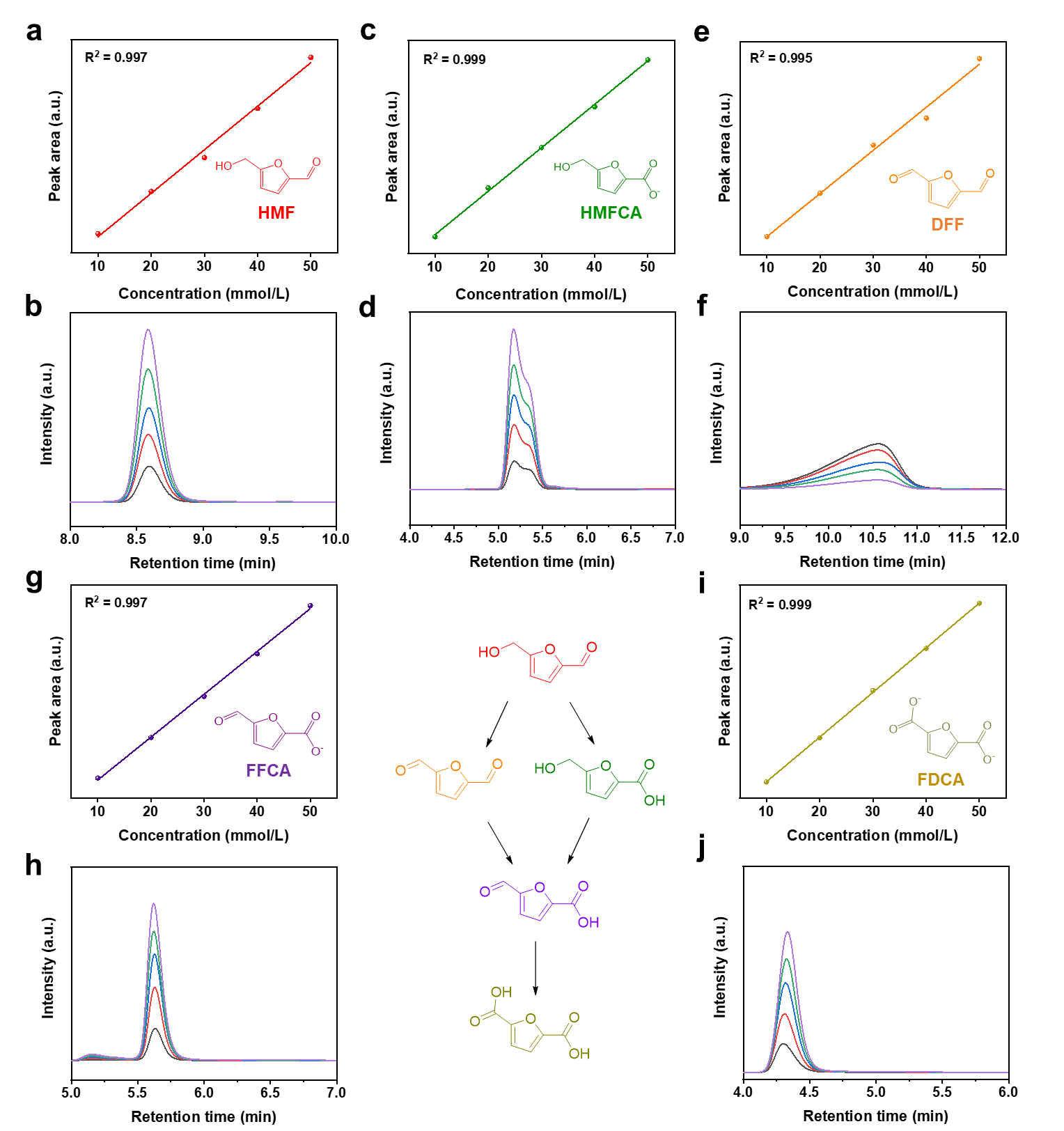
**

**Supplementary Figure 15** Reference HPLC chromatogram traces and calibration curves for HMF, FDCA, HMFCA, DFF and FFCA.

**
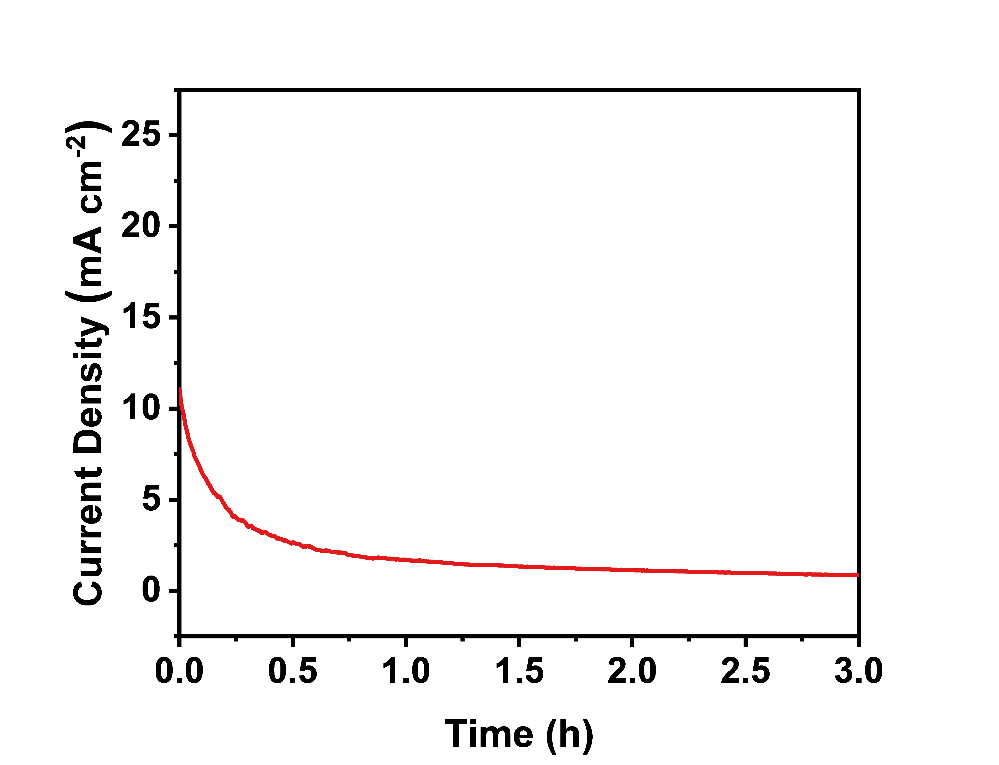
**

**Supplementary Figure 16** Long time i-t curve of HMF oxidation reaction at 0.43 V.


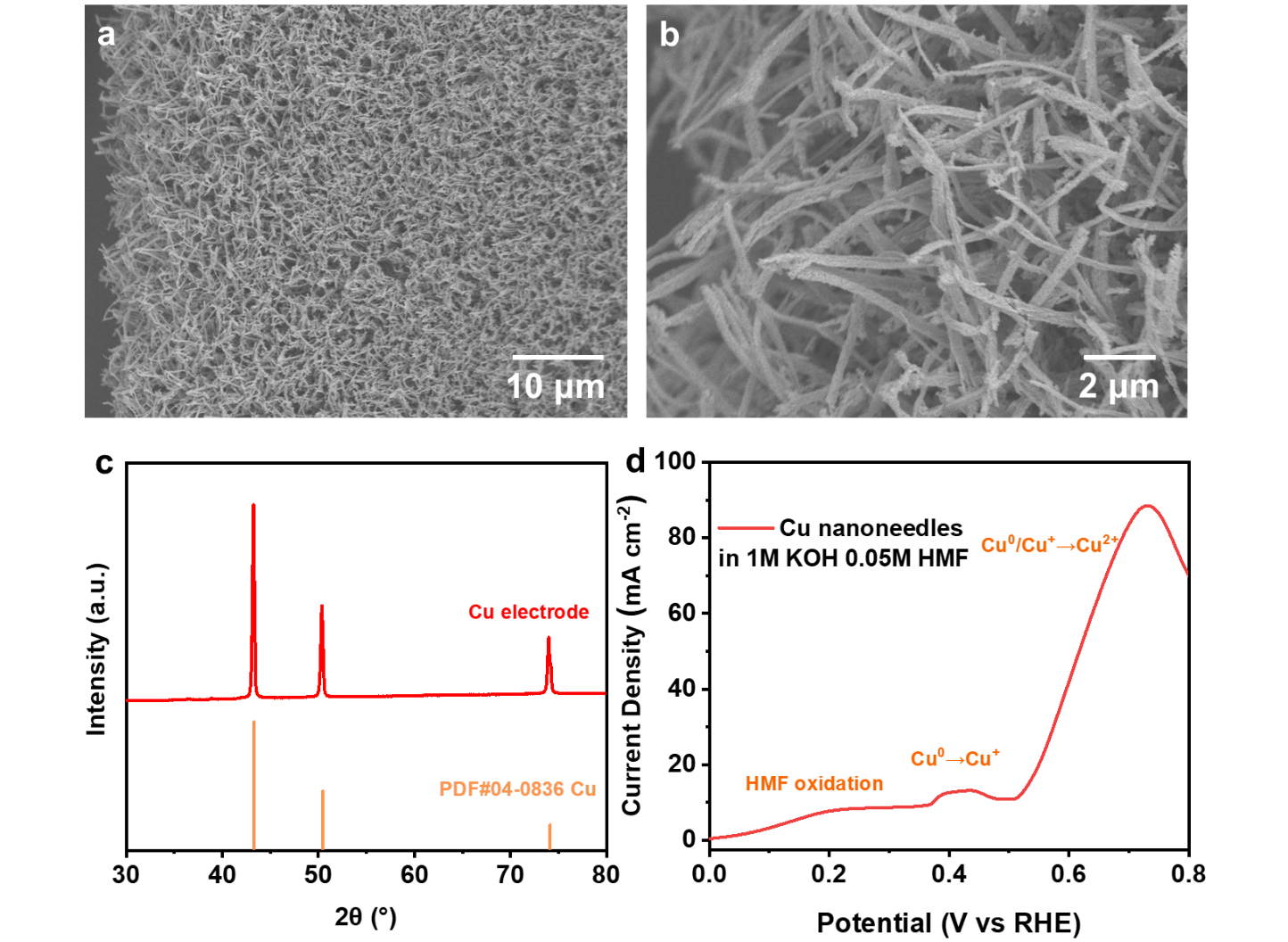


**Supplementary Figure 17** the SEM images of as synthesis Cu nanostructure electrode in low (a) and high (b) magnification. (c) The XRD pattern of Cu nanostructure electrode. (d) The LSV curve of Cu nanostructure electrode in 1 M KOH and 0.05 M HMF.

As shown in **Supplementary Fig.17 a** and **b**, the surface of the Cu foam is covered by Cu nanoneedles with a diameter of 200 nm. Such a nanostructure with a large surface area is beneficial to both electrocatalysis activity and the surface-enhancing Raman effect. **Supplementary Fig.17c** shows the XRD pattern of the Cu electrode, in which the peaks at 43.3°, 50.4°, and 74.0° are assigned to Cu (PDF#04-0836). **Supplementary Fig.17d** shows the electrochemical behavior of the Cu nanostructure electrode in 1M KOH and 0.05 M HMF. It clearly shows that the HMF hydrogen production reaction occurs at 0–0.5 V, whereas the partial Cu is oxidized to Cu_2_O at 0.5–0.6 V. However, both Cu and Cu_2_O are oxidized to Cu(OH)_2_ at a potential above 0.6 V.


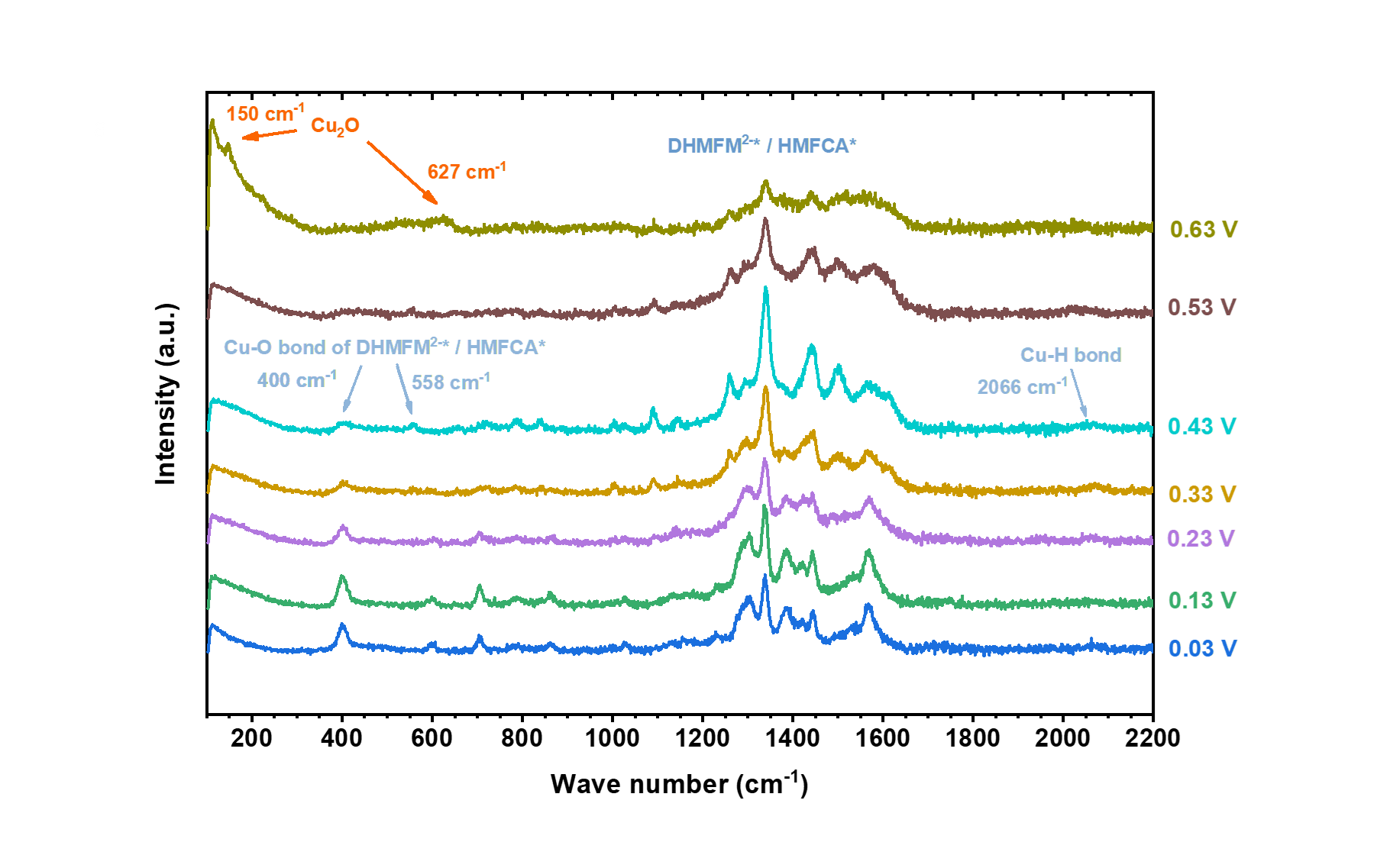


**Supplementary Figure 18** In situ Raman spectra by Cu nanostructure electrode in 1 M KOH and 0.05 M HMF.

An in-situ Raman experiment is conducted on the Cu nano-structure electrode, and the results are shown in **Supplementary Fig. 18**, which is different from the spectra conducted on the gold nano-structure electrode. The main difference lies in the signals for the intermediates HMFDM^2-^ and HMFDM^-^. In the *in-situ* Raman experiment for the Cu nano-structure electrode, the peak at 400 cm^-1^ is attributed to the Cu-O of HMFDM^2-^ absorbed species. The other typical Cu-O peaks, which have been widely studied, can be assigned to Cu_2_O (150, 220, 415, 520, 630 cm^-1^), CuO (303, 350, 636 cm^-1^), Cu(OH)_2_ (292, 488 cm^-1^) and CuO_2_^-^ (636 cm^-1^). (*ACS Catal. 2016*, **6**, 2473–2481) However, there is no Cu-O peak for HMFDM^-^ observed, indicating that no HMFDM^-^ pathway occurs on the surface of the Cu electrode. The observation is in line with the performance results. In addition, The Cu-H peak, which is considered the most important intermediate for the production of H_2_, is also observed at 2066 cm^-1^. When the potential rises to 0.33V-0.43V, some new peaks appear, and the new peak at 558 cm^-1^ is attributed to the Cu-O of HMFCA absorbed species. Notably, the intensities of HMFDM^2−^ and HMFCA peaks decrease since the Cu electrode is oxidized to Cu_2_O above 0.53 V and Cu_2_O is not active in hydrogen production from aldehyde. Meanwhile, the peaks of Cu-H at 2066 cm^-1^ disappear at this point, confirming the stoppage of hydrogen production from aldehyde. As a result, DHMFM^2-^ is the main intermediate of the Cu electrode-catalyzed aldehyde hydrogen production reaction, which not only explains why the selectivity of H_2_ is close to 100% on Cu but also verifies the purposed mechanisms.
